# 

**Published:** 1915-04

**Authors:** 


### "Red Back"

LIBRARY
APR. 22 1915
SURGEON GENERAL'S OFFICE

ESTABLISHED JULY, 1885

PUBLISHED MONTHLY AT AUSTIN, TEXAS, BY

MRS. F. E. DANIEL, - - - Publisher and Managing Editor

Subscription, $1.00 a Year, in Advance. - - - - - Single Copies, 10 Cents

Entered at the Postoffice at Austin, Texas, as second class mail matter.

